# Survival after laparoscopic and open surgery for colon cancer: a comparative, single-institution study

**DOI:** 10.1186/s12893-015-0013-5

**Published:** 2015-03-25

**Authors:** Fabio Cianchi, Giacomo Trallori, Beatrice Mallardi, Giuseppe Macrì, Maria Rosa Biagini, Gabriele Lami, Giampiero Indennitate, Siro Bagnoli, Andrea Bonanomi, Luca Messerini, Benedetta Badii, Fabio Staderini, Ileana Skalamera, Giulia Fiorenza, Giuliano Perigli

**Affiliations:** Center of Oncological Minimally Invasive Surgery (COMIS), Department of Surgery and Translational Medicine, University of Florence, Italy Largo Brambilla 3, 50134 Florence, Italy; Department of Experimental and Clinical Biomedical Sciences, University of Florence, Florence, Italy; ISPO, Florence, Italy; IFCA, Florence, Italy; Unit of Gastroenterology, AOU Careggi, Florence, Italy; Department of Experimental and Clinical Medicine, University of Florence, Florence, Italy

**Keywords:** Colon cancer, Laparoscopic surgery, Survival, Lymph nodes

## Abstract

**Background:**

Some recent studies have suggested that laparoscopic surgery for colorectal cancer may provide a potential survival advantage when compared with open surgery. This study aimed to compare cancer-related survivals of patients who underwent laparoscopic or open resection of colon cancer in the same, high volume tertiary center.

**Methods:**

Patients who had undergone elective open or laparoscopic surgery for colon cancer between January 2002 and December 2010 were analyzed. A clinical database was prospectively compiled. Survival analysis was calculated by using the Kaplan-Meier method.

**Results:**

A total of 460 resections were performed. There were no significant differences between the laparoscopic (n = 227) and the open group (n = 233) apart from tumor stage: stage I tumors were more frequent in the laparoscopic group whereas stage II tumors were more frequent in the open group. The mean number of harvested lymph nodes was significantly higher in the laparoscopic than in the open group (20.0 ± 0.7 *vs* 14.2 ± 0.5, P < 0.01). The 5-year cancer-related survival for patients undergoing laparoscopic resection was significantly higher than that following open resections (83.1% *vs* 68.5%, P = 0.01). By performing a stage-to-stage comparison, we found that the improvement in survival in the laparoscopic group occurred mainly in patients with stage II tumors.

**Conclusions:**

Our study shows a survival advantage for patients who had undergone laparoscopic surgery for stage II colon cancer. This may be correlated with a higher number of harvested lymph nodes and thus a better stage stratification of these patients.

## Background

Laparoscopic surgery for colon cancer is now practiced widely, with proven short-term benefits for patient recovery. Several studies have shown that it is possible to have reduced hospital stay, earlier return of bowel function, better pulmonary function and reduced morbidity in comparison with open surgery [1-5]. At the beginning of the laparoscopic era, some concerns were raised regarding the oncological outcome of this approach in the treatment of colorectal cancer. However, data from randomized trials and meta-analyses have definitively established that laparoscopic colonic surgery is at least equivalent to open surgery [2,6-9].

Whether the advantage of fewer complications and better short-term outcomes can be translated into better patient survival is controversial. Lacy *et al.* [10] have recently reported some unexpected, positive results regarding long-term survival of patients submitted to laparoscopic colon cancer resection within a randomized, controlled trial. These authors demonstrated a significantly higher tumor-related survival in patients who had undergone laparoscopic surgery when compared with open surgery, and this survival advantage was more pronounced in patients with stage III tumors. Other reports, although from retrospective, non-randomized studies, are in line with these results [11-14], showing potential survival benefits for patients undergoing laparoscopic surgery compared with historical series of conventional open surgery.

A number of hypotheses have been proposed to explain this beneficial oncological role of laparoscopic surgery in the treatment of colon cancer. In particular, laparoscopic resection is known to attenuate the surgical stress and systemic inflammatory response following surgery when compared with open surgery [15-17]. As a consequence, the postoperative immune function is better preserved with laparoscopic surgery, which may lead to a significant increase in the patient’s resistance to cancer.

The purpose of this study was to compare the cancer-related survival of patients undergoing laparoscopic and open surgery for non-metastatic colon cancer in the same, high volume tertiary center.

## Methods

### Patients

A retrospective cohort study was performed by comparing patients undergoing laparoscopy to those undergoing open resection for colon cancer (at least 15 cm above the anal verge) with curative intent between January 2002 and December 2010 at the Center of Minimally Invasive Oncological Surgery, University of Florence, Italy. Cases were identified through a prospectively maintained database. During the study period, from 2002 to 2005, the choice of surgical approach was decided mainly by surgeons’ preference and patients’ choice. During this period, most operations were performed by three colorectal staff surgeons. Two performed both laparoscopic and open surgery, while the other performed only open operations. With the progressive maturation of laparoscopic techniques, laparoscopic surgery has been offered to all suitable patients since 2005. Postoperative complications were defined according to the Clavien-Dindo classification [18]. All patients were followed postoperatively according to a protocol which includes physical examination, serum carcinoembryonic antigen determination, abdominal ultrasonography or computed tomography, and chest x-ray every 6 months. Total colonoscopy was performed every year. All patients were thoroughly informed about the study and gave written consent for the investigation. The study which was in full compliance with the Declaration of Helsinki, was approved by the ethics committee of Careggi University Hospital.

### Surgical technique

Surgery was performed with curative intent for all the patients in this study. Therefore, patients with residual macroscopic tumor after surgery, a secondary neoplasia or distant metastases were excluded from the study. Patients who had been converted to open surgery, mainly for huge, locally advanced lesions, or those with tumors associated with familial adenomatous polyposis or inflammatory bowel diseases, were also excluded. All laparoscopic procedures were performed through a standardized medial-to-lateral approach as previously described [19]. Briefly, this approach begins with proximal ligation of vascular pedicles, subsequent medial-to-lateral exploration of the retroperitoneum for identification and protection of important structures (e.g., duodenum, ureter), followed by mobilization and resection of the bowel with anastomosis. Dissection was performed in the majority of patients by ultrasonic dissectors. The specimen was extracted through an incision at a convenient site in the abdominal wall, protected by a wound protector, just large enough to allow specimen extraction. In case of proximal tumors, anastomosis was performed either intra- or extracorporeally. A left-sided or rectal anastomosis was performed using a circular stapler which was inserted transanally. Open resections were performed through a midline incision in a standard manner.

Operative techniques, standardized for open and laparoscopic surgery at our institution, included lymphadenectomy according to tumor location as previously described [19]. Briefly, for proximal tumors, (i.e., located up to the splenic flexure) we performed right hemicolectomy extended to the mid-transverse colon with lymphadenectomy at the origin of the ileocolic, right colic, and middle colic arteries when necessary. We performed left hemicolectomy *plus* high anterior rectal resection for distal tumors (i.e., located from the splenic flexure to the rectal-sigmoid junction) and lymphadenectomy was extended to the origin of the inferior mesenteric vessels. A distal clearance of at least 2 cm of healthy mucosa from the lower edge of the tumors was provided in all cases.

### Pathological examination

Pathology staff and examination technique of surgical specimens did not change during the entire period of study. All the surgical specimens were fixed in 10% formalin solution and routinely processed for paraffin embedding. Tumor stage was determined according to the sixth edition of the American Joint Committee of Cancer (AJCC) staging system [20]. The number of examined lymph nodes (LNs) was ascertained by reference to the histopathology report of each patient. Lymph nodes were identified in the mesocolic fat of the surgical specimens by sight and palpation. Routine histologic examination was performed using hematoxylin and eosin staining. Histologic processing of the specimens was the same for all patients. No special fat clearance or staining techniques were employed. The following histopathological features were assessed for each tumor specimen: tumor type (classified as adenocarcinoma or mucinous carcinoma if more than 50% of the tumor volume was composed of mucin) and tumor differentiation (only for adenocarcinomas, classified as well, moderately or poorly differentiated).

### Statistical analysis

Cancer-related survival was calculated from the date of the operation to the date of death due to cancer in patients who had curative surgery. Alive patients, with or without evidence of recurrent disease or lost to follow-up were censored at the date last known to be alive. Patients without evidence of recurrence at death were censored at the date of death. The analysis was performed on a treatment-received basis when comparing data on patients undergoing either laparoscopic or open resection. Categorical variables within laparoscopic and open groups were compared using Fisher’s exact test or the chi-square test. Quantitative variables were summarized by mean and SEM or median and range. Groups were compared using the Mann-Whitney test. Differences in survival between groups were compared using Kaplan-Meier curves and tested with the log rank test. Statistical significance was considered if P < 0.05. STATA Statistical Software release 6.0 (College Station, TX, USA) was used for all the analyses.

## Results

Between January 2002 and December 2010, 460 patients underwent curative resection for non-metastatic colon cancer. A total of 227 resections (49.3%) were performed by the laparoscopic approach, whereas 233 patients (50.7%) underwent open resections. All operations were performed in an elective setting and patients with emergency operations were excluded. All patients with stage III tumors underwent adjuvant chemotherapy. Five patients (1.0%), 3 in the open and 2 in the laparoscopic group, were lost to follow-up 2 years after the operation. The median length of follow-up after laparoscopic resection was 42.0 months (range, 3-120) compared with 50.0 months (range, 4-120) after open resection.

The groups of patients who underwent open and laparoscopic surgery did not significantly differ according to gender and age (Table 1). The operative mortality rate was 1.7% in the open and 1.3% in the laparoscopic group (P = 0.7). The incidence of postoperative complications was significantly lower after laparoscopic resection than after open surgery (Table 1). The median length of postoperative stay was significantly shorter at 6 days following laparoscopic surgery compared with 9 days following open resection (Table 1). There was no significant difference between the two groups in terms of tumor site, histotype or differentiation (Table 1). Stage I tumors were more frequent in the laparoscopic than in the open group whereas stage II tumors were more frequent in the open than in the laparoscopic group (Table 1). The overall average number of examined LNs per case was significantly higher in the laparoscopic than in the open group (Table 2). This result was also confirmed when the number of examined LNs was compared between each TNM stage category (Table 2). There was no significant difference in the number of positive LNs for stage III tumors between the two groups (Table 2).

**Table 1:** **Comparison of clinical outcomes between patients of the open and laparoscopic groups**

	**Open resection (n =233)**	**Laparoscopic resection (n = 227)**	**P**
**Gender** (M/F)	122/111	117/110	0.8*
**Mean age** (yr)	68.3 ± 0.9	70.0 ± 0.7	0.08^**§**^
**Operative Mortality (30 days)**	4 (1.7)	3 (1.3)	0.7*
**Overall postoperative complications (%)**	41 (17.6)	16 (7.0)	<0.01*
**Total grade I**	5 (2.2)	0	
Wound infection	5	0	
**Total grade II**	24 (10.3)	9 (4.0)	
Urinary retention/infection	1	0	
Arrhythmia	5	4	
Pneumonia	4	2	
Ileus	10	1	
Deep vein thrombosis	3	0	
Cerebrovascular accident	1	1	
Pancreatitis	0	1	
**Total Grade III**	11 (4.7)	6 (2.6)	
Intestinal obstruction	4	1	
Gastrointestinal bleeding	3	2	
Anastomotic leak	4	3	
**Total Grade IV**	1 (0.4)	1(0.4)	
Cardiac failure	1	1	
**Median hospital stay (days)**	9 (5-16)	6 (3-10)	<0.05^**§**^
**Tumor site (%)**			0.9*
Proximal tumors	130 (56.8)	127 (56.0)	
Distal tumors	103 (44.2)	100 (44.0)	
**Tumor type (%)**			0.7*
Adenocarcinoma	190 (81.5)	182 (80.2)	
Mucinous carcinoma	43 (18.5)	45 (19.8)	
**Tumor differentiation (%)**			0.3°
Well differentiated	25 (13.1)	16 (7.0)	
Moderately differentiated	152 (65.2)	155 (68.2)	
Poorly differentiated	13 (5.5)	11 (4.9)	
**Tumor stage (%)**			<0.01°
I	35 (15.0)	64 (30.7)	
II	123 (45.8)	82 (35.1)	
III	75 (35.9)	81 (34.1)	

*Fisher’s exact test.

^**§**^Mann-Whitney test.

°Chi-square test.

**Table 2:** **Analysis of lymph node harvest between patients of the open and laparoscopic groups**

	**Open resection (n =233)**	**Laparoscopic resection(n = 227)**	**P**
**Harvested lymph nodes (total)**	14.2 ± 0.5	20.0 ± 0.7	<0.01^**§**^
Stage I	11.8 ± 1.0	18.1 ± 1.5	0.01^**§**^
Stage II	14.4 ± 0.7	21.8 ± 1.1	<0.01^**§**^
Stage III	14.9 ± 0.9	19.8 ± 1.0	<0.01^**§**^
**Involved lymph nodes**	3.9 ± 0.3	3.3 ± 0.8	0.6^**§**^

^**§**^Mann-Whitney test.

**Figure 1:**
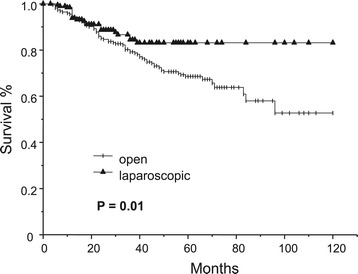
**Comparison of cancer-related survival of patients who underwent laparoscopic and open surgery for colon cancer.**

**Figure 2:**
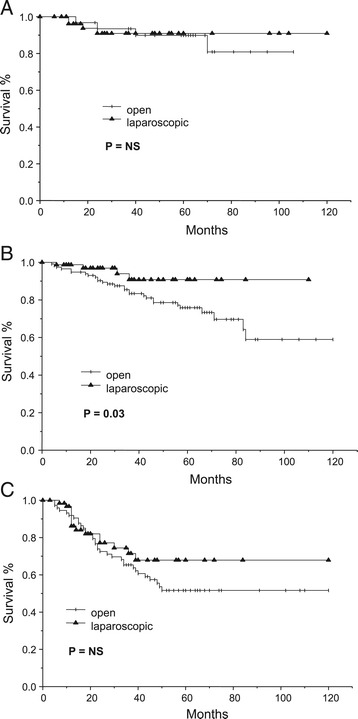
**Comparison of cancer-related survival of patients who underwent laparoscopic and open surgery according to tumor stage. A)** Comparison of cancer-related survival of patients who underwent laparoscopic and open surgery for stage I colon cancer. **B)** Comparison of cancer-related survival of patients who underwent laparoscopic and open surgery for stage II colon cancer. **C)** Comparison of cancer-related survival of patients who underwent laparoscopic and open surgery for stage III colon cancer.

The 5-year cancer-related survival for patients undergoing laparoscopic resection was significantly higher than that for patients receiving open resections (83.1% vs 68.5%, P = 0.01) (Figure 1). The comparison of survival of patients with stage I, II and III diseases is shown in Figures 2A,B and C, respectively. There was no difference in survival in patients with stage I disease. The improvement in survival in the laparoscopic group occurred mainly in patients with stage II tumors: the 5-year survival rates were 75.8% and 90.7% for the open and laparoscopic groups, respectively (P = 0.03). There was also a trend towards better survival in patients with stage III tumors, although it did not show any statistical significance.

## Discussion

Laparoscopic technique has revolutionized the treatment of colorectal malignancy in recent years. Randomized controlled trials have clearly shown that laparoscopy provides favorable operative outcomes in terms of less pain, quick recovery of the gastrointestinal tract, a shorter hospital stay and cosmetic satisfaction when compared with open surgery [2,3,6]. Moreover, a recent meta-analysis [21] and two large retrospective studies [22,23] which included a large number of patients, were also able to demonstrate a significant reduction in mortality rate and lower morbidity after laparoscopic resection.

Survival is the most important outcome for assessing treatment success for malignant disease. Three major, randomized trials have shown that laparoscopic resection can produce an equivalent oncological outcome to that achieved with open surgery but did not identify a survival advantage in favor of laparoscopy [2,6,7]. In a single-center randomized study, Lacy *et al*. [10] have shown a cancer-related survival advantage after laparoscopic surgery for stage III colon cancer. Capussotti *et al*. [24] also found that laparoscopic resection was associated with significantly better disease-free and cancer-related survival in patients with stage III colon cancer. Other groups have recently reported superior survival for patients undergoing laparoscopic resection, even for those with stage II colorectal cancer [25]. All these studies have distinguished between colon and rectal cancer in their survival analysis, showing an improvement in survival after laparoscopic resection mostly in the colon cancer patients.

In the present study, we found overall better survival for patients who had undergone laparoscopic resection for colon cancer. However, the study is retrospective and the difference in follow-up period between the two study groups as well biases in the selection of patients were unavoidable and might affect our survival analysis. In particular, the two groups of study patients were not homogeneous regarding tumor stage: stage I tumors were more frequent in the laparoscopic group whereas stage II tumors were more frequent in the open one. As a consequence, a stage-to-stage comparison was made between the two groups of patients. Interestingly, we found that improvement in survival was limited to patients undergoing laparoscopic resection for stage II tumors.

One of the reasons accounting for this better survival might be the difference in LN harvesting between the two groups of patients. Indeed, we found that the number of retrieved and examined LNs was significantly higher in patients who had undergone laparoscopic resection. This finding is particularly relevant if we consider that the mean values of LNs found in our patients, both in the open and laparoscopic group, are among the highest reported in the literature and higher than the threshold value of 12 LNs recommended by the AJCC [26]. It is well known that appropriate lymphadenectomy during colorectal cancer surgery is crucial for the patient’s oncological outcome for at least two reasons. First, it reduces the risk of residual nodal disease, and second, only the examination of a large number of LNs can predict accurate nodal staging. Our finding of improved survival only in patients with stage II, i.e. T_3_N_0_, colon cancer underlines the importance of an adequate number of harvested LNs so as to reduce the risk of overlooking one or more metastatic LNs in the surgical specimens. Therefore, better stratification of our stage II patients within the laparoscopic group may explain the survival advantage when compared with the open surgery patients.

Recently, similar results have been reported by Law *et al.* [25]. Among their 814 patients who had undergone laparoscopic resection for both colon and rectal cancer, they found an improvement in survival only in patients with stage II disease in comparison with the open group patients (n = 1197). Even in this series of patients, there were significantly more LNs examined in the laparoscopic than in the open group (median value, 13 vs 11 in the two groups, respectively).

As already discussed in a previously published study [19], we do not have a clear explanation for the better accuracy in LN retrieval after laparoscopic resection. Some authors have stated that laparoscopy for colorectal cancer offers the opportunity for a meticulous dissection of the mesocolon and mesorectum under direct vision while facilitating an accurate lymphadenectomy [27,28]. However, other factors may influence the number of examined LNs in cancer specimens. The experience of the surgeon performing the operations and the skill of the pathologist in retrieving LNs are considered the most important among these factors [29-31]. In our study, the majority of open procedures were performed by one surgeon skilled in gastrointestinal surgery and the same operative techniques, especially the same types of lymphadenectomy, were exactly reproduced laparoscopically by the other two surgeons. Histopathologic examination remained uniform during the study with no changes in the pathologic team or in LN harvesting technique. One possible explanation is that the prognostic importance of the number of retrieved LNs in colorectal cancer specimens was emphasized by the AJCC [26] only at the beginning of the 2000s and thus, the increase in the number of harvested LNs in our more recent laparoscopic experience may be due to a greater effort by both the surgeons and the pathologists to remove and examine the maximum number of LNs.

Other reasons might account for the better survival observed in patients who underwent laparoscopic surgery for colon cancer. One of the most important of these might be the prompt preservation of the patient’s immunological response against cancer from the first postoperative days [32]. This is mainly due to the reduced inflammatory stress and thus the reduced inhibition of cell-mediated immunity observed after laparoscopic surgery [15]. Moreover, a number of studies have demonstrated that cancer-prone cytokines, such as interleukin 6 and vascular endothelial growth factor, are produced significantly more after open than laparoscopic surgery [16,33]. Altogether, these data might help to explain our better oncologic outcome after laparoscopic resection.

## Conclusions

Within the limitations of a retrospective analysis, our study shows a survival advantage for patients undergoing laparoscopic surgery for stage II colon cancer. This may be correlated with a higher number of harvested/examined LNs and thus a better stage stratification of these patients when compared with the open group. However, other reasons, such as a more efficient postoperative immune response, might be involved.

## Abbreviations

AJCC: American Joint Committee of Cancer
LN: Lymph node
